# Lyme neuroborreliosis presenting as rapidly progressive dementia - a case report

**DOI:** 10.1192/j.eurpsy.2025.1763

**Published:** 2025-08-26

**Authors:** S. Verhnjak

**Affiliations:** University Psychiatric Clinic Ljubljana, Ljubljana, Slovenia

## Abstract

**Introduction:**

Lyme borreliosis is one of the most common vector diseases transmitted by tick bites; it is caused by Borrelia burgdorferi. Mostly it manifests on the skin, in the nervous system or joints. It involves the nervous system in 10-15% of cases, of which 2-4 % affect the central nervous system. The most common manifestation is encephalitis, which has a diverse clinical picture.

**Objectives:**

We aim to describe a rare case and discuss the diagnostic challenges of a rapidly progressive disease.

**Methods:**

A detailed description of the patient based on our interview and clinical findings, including blood work, imaging, microbiological testing, lumbar puncture, and treatment.

**Results:**

A 67-year old female patient came to the psychiatric emergency room in March 2024, because of persistent anxiety and unexplained somatic disorders, including weight loss, tremor and unstable gait, which began a few months ago. She had some somatic diagnostic procedures done, with no abnormal findings. 2 weeks before being admitted she was sent to the emergency neurological unit because of fatigue and tremor. They excluded focal neurological signs and concluded that she had an adjustment disorder and suggested psychiatric treatment. In March 2024 she was admitted to the geriatric psychiatry ward, where at first our main differential diagnosis was pseudodementia. In the next few days her condition worsened. She appeared psychotic, with ideas of persecution and reference. On the psychological exam she had moderate cognitive decline with a focus on impaired attention, memory and executive systems, misinterpretations of past and current events, misidentifications of people and possible complex visual hallucinations. At that time we suspected she might have prolonged delirium. Because of an uncommon clinical picture, we pursued further diagnostics. The lumbar puncture showed cerebral spinal fluid (CSF) pleocytosis, which confirmed the diagnosis of encephalitis. Blood tested for multiple infectious causes was positive for Lyme borreliosis. The brain CT scan showed an inflammatory or infiltrative process in both cerebral hemispheres. We then transferred her to the infectious disease clinic where she had a brain MRI with contrast and her CSF was tested for Borrelia and other possible causes. On the MRI they suspected she had rhombencephalitis with leptomeningitis. After the diagnosis of neuroborreliosis was confirmed, she received a 4-week parenteral treatment with ceftriaxone. Two months after completing the treatment she has fully recovered.

**Conclusions:**

When faced with a patient with rapidly progressive dementia a wider range of possible diagnoses has to be considered. We have to be aware of the importance of recognising the cause of the disease sooner, as the patient may have a treatable condition.

**Disclosure of Interest:**

None Declared

